# C/T Ratios in Human Eyeblink Conditioning Paradigms to Examine Cerebellar Function and ADHD: A Narrative Review

**DOI:** 10.3390/bs16010111

**Published:** 2026-01-13

**Authors:** Caleb S. Spink, John W. Walker, Shane H. Phillips, John Michael Falligant

**Affiliations:** Department of Psychological Sciences, Auburn University, Auburn, AL 36849, USA; css0121@auburn.edu (C.S.S.); jww0086@auburn.edu (J.W.W.); szp0199@auburn.edu (S.H.P.)

**Keywords:** attention-deficit/hyperactivity disorder, animal models, cerebellum, C/T ratio, eyeblink conditioning, Pavlovian

## Abstract

Eyeblink conditioning (EBC), a form of Pavlovian learning that relies on cerebellar circuits, offers a translationally relevant assay of adaptive learning and cerebellar integrity. In delay EBC, a conditioned stimulus (CS), such as a tone, overlaps with and co-terminates with the unconditioned stimulus (US), typically a brief air puff to the cornea. Trace EBC introduces a stimulus-free interval between CS offset and US onset, engaging additional brain structures such as the hippocampus. Acquisition of conditioned responses (CRs), their timing, and resistance to extinction have all been linked to cerebellar function. While EBC is a well-established paradigm in the experimental analysis of behavior and neuroscience, human studies applying it to ADHD populations remain limited and show inconsistent findings. One potential explanation for this variability lies in procedural differences across studies, particularly in the temporal structure of conditioning trials. A key parameter in Pavlovian learning is the ratio of the inter-US interval (C; time between USs) to the CS–US interval (T; time between CS onset and US onset). Known as the C/T ratio, this value indexes the informational value of the CS in predicting the US and has been shown to influence acquisition speed and response strength. Despite its theoretical importance, the C/T ratio is rarely reported or standardized in human EBC studies involving ADHD. The present review aims to characterize procedural features—especially C/T ratios—used in EBC research with ADHD populations or models, with a focus on how such parameters may shape performance and interpretability in studies probing cerebellar function.

## Simple Summary

Eyeblink conditioning (EBC) is an established paradigm for studying Pavlovian learning that is dependent on the cerebellum. Some ADHD symptoms are caused by cerebellar dysfunction. Preliminary evidence suggest EBC shows promise in studying cerebellar dysfunction in people diagnosed with ADHD. However, the C/T ratio—defined as the inter-US interval divided by the CS–US interval—remains unstandardized in this research area, and inconsistencies in this parameter may partly explain the mixed findings observed to date, in addition to strain-related differences documented in animal studies.

## 1. Introduction

Attention-deficit/hyperactivity disorder (ADHD) is a neurodevelopmental condition marked by persistent inattention, hyperactivity, and impulsivity. While the disorder has traditionally been associated with dysfunction in prefrontal and striatal circuits, mounting evidence implicates the cerebellum as a key contributor to its neurobiological underpinnings. Structural neuroimaging studies have revealed reduced cerebellar volume in individuals with ADHD, particularly in the posterior inferior vermis—an area associated with attention and emotional regulation (Mostofsky et al., 1998; Bledsoe et al., 2011). Additionally, reduced functional connectivity between the cerebellum and the ventrolateral prefrontal cortex (VLPFC) has been observed, further highlighting the cerebellum’s role in modulating cognitive processes disrupted in ADHD (Wolf et al., 2009).

One promising method for probing cerebellar function is eyeblink conditioning (EBC), a paradigm that offers a sensitive, translationally relevant assay of Pavlovian learning and cerebellar integrity. In delay EBC, a conditioned stimulus (CS), such as a tone, precedes and overlaps with an unconditioned stimulus (US), such as an airpuff to the eye, until the two co-terminate. In trace EBC, a stimulus-free interval separates the offset of the CS from the onset of the US, engaging additional brain regions, particularly the hippocampus (Clark et al., 2001; Tseng et al., 2004). Acquisition of conditioned responses (CRs), their temporal precision, and resistance to extinction are all reliably linked to cerebellar function (Garcia et al., 1999), with trace conditioning also implicating hippocampal contributions (Halverson et al., 2018).

**Figure 1:**
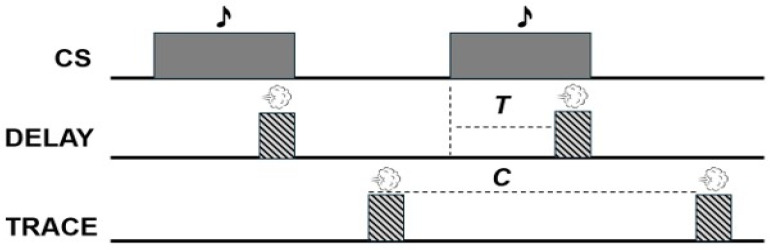
A diagram visualizing the difference between delay and trace eyeblink conditioning (EBC) with a tone serving as the conditioned stimulus (CS). The airpuff (US) will overlap and co-terminate with the CS during delay paradigms while trace EBC features a gap between the termination of the CS and US.

Given its well-mapped neural substrates, EBC has been widely used in both human and nonhuman models to investigate learning and timing processes across neurodevelopmental conditions—including autism and dyslexia—but remains underutilized in ADHD research (Reeb-Sutherland & Fox, 2015). This is notable, as structural and functional cerebellar abnormalities are well documented in ADHD, and EBC provides a noninvasive, behaviorally grounded method of assessing cerebellar function. Significant results may manifest as different percentages of trials with conditioned responses, timing of conditioned response emission, or response force determined by recorded amplitude or velocity of the reflex. This conditioning procedure can shed light on the temporal dimensions of learning in a wide range of individuals and provide information on how learning occurs. Like most Pavlovian procedures, EBC can be divided into delay and trace tasks. Again, in delay EBC, the US is presented while the CS is active and the two co-terminate while trace EBC includes a gap between the cessation of the CS and US (Clark et al., 2001; Figure 1). Despite differences in experimental arrangement, both delay and trace EBC are dependent upon cerebellar functioning (Halverson et al., 2018).

The processes underlying delay eyeblink conditioning have been well documented, allowing for the entire circuit to be mapped. There are two pathways that are involved in expressing a CR. The pontine nuclei (PN) receive information about the CS from the vestibulocochlear nucleus. The PN then transmits this information to granule cells (GC) as well as the interpositus nucleus (IPN) within the cerebellar cortex. The GC sends signals to Purkinje cells (PC) and inhibitory interneurons. The dorsal accessory inferior olive (DAO) receives information about the US from the trigeminal nucleus in the brainstem. The axons of DAO neurons form climbing fibers and send projections of the information to the IPN and PC. The IPN combines the excitatory signals from the PN and DAO with inhibitory input from the PC and outputs this information to the red nucleus (RN). To express a CR, the RN activates both the accessory facial nucleus and accessory abducens nucleus. Before conditioning occurs, the excitatory cells are overcome by the inhibitory signals from the PC, but the UR is still expressed through the trigeminal nucleus. As CS-US pairings are repeated, there is decreased firing from the PC and increased firing from IPN neurons. Though the differences in the trace and delay conditioning procedures are minimal, learning in trace conditioning depends on regions in the forebrain, such as the hippocampus, in addition to the aforementioned cerebellar circuit (Takehara-Nishiuchi, 2018).

Damage to the anterior lobe of the cerebellum has been demonstrated to inhibit acquisition in conjunction with lesions to the anterior interpositus nucleus (Garcia et al., 1999) prevent extinction of delay-conditioned eyeblinks (Perrett & Mauk, 1995) and contributes to CR timing and amplitude (Garcia & Mauk, 1998). Damage to other areas of the cerebellum has been documented to reduce the percentage of conditioned responses as well as onset latency (Gerwig et al., 2005). Cerebellar volume is also correlated with an increase in the percentage of conditioned responses (Woodruff-Pak et al., 2000). In contrast to delay EBC, the hippocampus has been implicated in trace EBC. Hippocampectomies reduce acquisition exclusively for trace paradigms (Tseng et al., 2004) and have the potential to disrupt acquisition depending upon CS-US duration (Moyer et al., 2015) while hippocampal lesions reduce CR latency and CR peak latency (Solomon et al., 1986; see Gerwig et al., 2007 for a review).

Despite the cerebellum’s documented involvement in ADHD, the limited EBC studies conducted with ADHD populations have produced mixed results. For example, some have reported no significant differences in EBC acquisition between ADHD and neurotypical participants using conventional short CS–US intervals (Coffin et al., 2005), whereas others observed group differences only when long CS–US intervals were employed (Frings et al., 2010). These inconsistencies may reflect variability in procedural parameters—particularly the temporal structure of trials—as well as differences in paradigm type (e.g., delay vs. trace EBC), which engage partially distinct neural circuits. Such heterogeneity not only complicates interpretation but may also obscure subtle deficits in cerebellar function. A necessary first step toward resolving these ambiguities is to characterize how EBC procedures have been implemented and reported across studies.

In Pavlovian learning, the C/T ratio refers to the ratio of the cycle time (C)—the average time between unconditioned stimuli (US)—to the trial time (T), or the interval between the onset of the conditioned stimulus (CS) and delivery of the US. This ratio reflects how much the CS reduces temporal uncertainty about the US, thereby increasing its predictive value. According to the Information-Theoretic model and Delay Reduction Theory, higher C/T ratios lead to faster and stronger acquisition of conditioned responses (Balsam & Gallistel, 2009; Shahan & Cunningham, 2015). For example, a C/T ratio of 40 (e.g., C = 20 s, T = 0.5 s) signals a much greater reduction in delay—and thus more learning—than a ratio of 2 (e.g., C = 4 s, T = 2 s). C/T ratios can been manipulated by increasing the ITI and/or the interstimulus interval (ISI), or the time between the presentation of the CS and US. Despite strong support from nonhuman models, this parameter has received little empirical attention in human EBC research, particularly in studies involving ADHD. However, the length of the ITI during EBC procedures has been documented to affect the percentage of trials with conditioned responses and latency of the conditioned response (Boneau, 1958; Boneau et al., 1956) and the length of the ISI affects the percentage of conditioned responses during eyeblink conditioning tasks (Kjell et al., 2018).

This brief review surveys human and nonhuman studies involving eyeblink conditioning in ADHD populations or models, with particular attention to how procedural parameters—including C/T ratios—have been arranged and reported. Our goal is not to re-evaluate the efficacy of EBC in differentiating clinical and control groups, but to systematically characterize procedural trends, identify gaps in methodological consistency, and lay the groundwork for optimizing EBC paradigms to probe cerebellar function in ADHD more sensitively and reproducibly.

## 2. Materials and Methods

**Figure 2:**
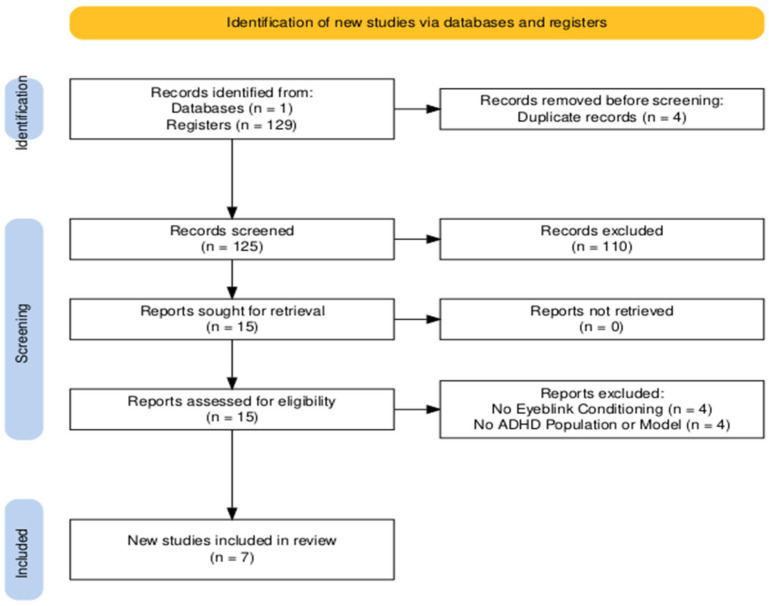
Out of the 125 non-duplicates, 110 were excluded during the abstract screening phase, and eight were excluded (four for not featuring eyeblink conditioning and four for lacking an ADHD population or model), leaving a total of seven for review. This diagram was made using PRISMA2020 software (Haddaway et al., 2022).

All articles were located within the Scopus database across multiple searches. Each search included forms of “eyeblink” and “conditioning” along with “ADHD” and terms associated with its viable models (i.e., “spontaneous hypertensive rat”, “dopamine transporter*”) in the title, abstract, and keywords. Results were limited to peer-review articles that were published in a peer-reviewed journal and written in the English language. The search consisted of abstract and full-text screening phases. An article progressed if it was or appeared to be: (1) an article that is or contains an original, empirical research report (2) features eyeblink conditioning, (3) included an ADHD population or suitable animal model, and if a human ADHD population was used (4) was published in or after 2015 to not overlap findings from Reeb-Sutherland and Fox (2015). Of the 125 articles, 110 were excluded during this phase. The remaining 15 articles were evaluated using the same criteria which led to the exclusion of eight articles with four not featuring EBC and four not including a suitable model of ADHD (Figure 2).

In the current review, we include studies involving both human participants and nonhuman animal models of ADHD, as the latter offer translational insights and experimental control over procedural variables. While not exhaustive, the following brief overview highlights key animal models relevant to ADHD and their potential application to cerebellar learning paradigms like eyeblink conditioning (EBC). The spontaneously hypertensive rat (SHR) remains the most widely accepted animal model of ADHD. Originally developed to study hypertension, SHRs reliably demonstrate core ADHD phenotypes, including hyperactivity, impulsivity, and deficits in sustained attention, relative to their progenitor control strain, the Wistar-Kyoto rat (WKY) (Adriani et al., 2003; Sagvolden et al., 2009). Other strains derived from WKY rats include WKHA (hyperactive) and WKHT (hypertensive) lines, with WKHA rats exhibiting attentional impairments (Chess et al., 2005), although some of these effects may be attributable to traits already present in the WKY background strain (Drolet et al., 2002). In addition to rat models, several mouse lines offer construct validity for ADHD-related traits. Dopamine transporter knockout (DAT-KO) mice, which lack the dopamine transporter gene, show pronounced hyperactivity and attentional deficits due to elevated extracellular dopamine levels (Adinolfi et al., 2019; Efimova et al., 2016). However, their relevance is debated, as individuals with ADHD often show increased dopamine transporter availability rather than reduced (Krause et al., 2003). Coloboma mutant mice, which lack part of chromosome 2 including the SNAP-25 gene, display hyperactivity and synaptic dysregulation linked to hyperactive ADHD subtypes (Hess et al., 1992; Brophy et al., 2002). Other models include thyroid hormone receptor β1 (TRβ1) mutants, which demonstrate hyperactivity, impulsivity, and inattention consistent with ADHD phenotypes observed in human patients with TRβ1 mutations (Siesser et al., 2006, Uter et al., 2020), and brain-derived neurotrophic factor (BDNF) knockout mice, which exhibit both hyperactivity and learning impairments (Linnarsson et al., 1997; Rios et al., 2001). Notably, clinical findings show that untreated individuals with ADHD may have elevated peripheral BDNF levels (Gumus et al., 2022; Shim et al., 2008), underscoring the complexity of translating molecular findings across species. These can additionally be sorted into hyperactive-impulsive for WKHA rats and Columba and BDNF-KO mice and SHR rats and DAT-KO and TRβ1 mutant mice for combined presentations; however, impulsive subtype models are not listed here and did not appear in the search. Although relatively few EBC studies have been conducted in these models, their behavioral and neurobiological profiles suggest promise for investigating cerebellum-dependent learning processes in ADHD. The inclusion of animal models in this review allows us to identify whether and how temporal parameters—particularly the C/T ratio—have been reported or manipulated across both human and nonhuman studies.

## 3. Results and Discussion

Although eyeblink conditioning (EBC) has been widely used to probe cerebellar function in neurodevelopmental disorders, its application to ADHD populations has been limited. Fewer than ten peer-reviewed studies have examined EBC in individuals with ADHD or suitable ADHD models, and these vary considerably in procedural design (Table 1). Across studies, key associative learning parameters—including the conditioned stimulus–unconditioned stimulus (CS–US) interval (T), the intertrial interval or inter-US interval (C), and the total number of acquisition trials—have not been standardized. This variability presents a major challenge for interpreting outcomes and drawing inferences about cerebellar involvement.

Among the studies reviewed, the CS–US interval (T) typically ranged from 300 to 1000 ms. Some studies employed relatively short intervals (e.g., 400 ms), which favor cerebellar-based learning, while others used extended CS–US intervals (e.g., 800–1000 ms) that may engage additional hippocampal processes (Table 2). Far less attention has been paid to the inter-US interval (C), which was often reported as a range (e.g., “15–25 s between trials”) or not at all. As a result, the C/T ratio, a parameter shown to strongly predict the rate and robustness of Pavlovian learning in both nonhuman and human paradigms (Balsam & Gallistel, 2009; Shahan & Cunningham, 2015), has not been explicitly considered in the design or interpretation of EBC studies in ADHD. Limited attention to C/T ratios may partly explain mixed findings in the literature. For instance, Frings et al. (2010) found impaired CR acquisition only when using long CS–US intervals—procedures likely associated with low C/T ratios—while other studies using shorter intervals failed to detect group differences (Coffin et al., 2005). Without controlling for or reporting the C/T ratio, it remains unclear whether such differences reflect genuine neurobehavioral effects or are an artifact of suboptimal conditioning parameters. To date, no human EBC study involving ADHD has systematically manipulated or explicitly documented the C/T ratio. This omission represents a significant gap, given theoretical and empirical work demonstrating that higher C/T ratios—those in which the CS provides a greater reduction in uncertainty or delay to the US—facilitate faster and more reliable acquisition of conditioned responses. As such, future work should prioritize explicit reporting and systematic manipulation of both C and T, enabling more direct tests of how these timing variables modulate associative learning in ADHD.

### Beyond the C/T Ratio

Although the C/T ratio is a key timing parameter, it operates alongside other sources of variability that can affect conditioning outcomes. In particular, sex and age systematically influence EBC performance and need to be controlled to reduce heterogeneity across studies. For example, Thanellou et al. (2009) found that male WKHA rats had lower conditioned response onset and peak latencies compared to male WKHT rats while there were no significant differences between the females. Additionally, it has been demonstrated that female SHRs require a greater number of trials to acquire tasks than male SHRs while this effect was not demonstrated across WKY rats (Bucci et al., 2008) as well as females exhibiting decreased habituation (Nunes et al., 2018). In the neurotypical human literature, females produce a greater percentage of conditioned responses than males (Löwgren et al., 2017). These results are likely applicable to ADHD populations, though no included or control articles mentioned effects of sex. Therefore, comparisons between ADHD and non-ADHD subjects should be restricted by sex for proper analysis of EBC effects.

As the cerebellum is not static, age (i.e., cerebellar development) has been demonstrated to impact performance on eyeblink conditioning tasks. Löwgren et al. (2017) found that adults (μ = 29.3, s = 8.6) had a higher percentage of CRs than children (μ = 8.8, s = 1.3) with older children outperforming younger children. Additionally, younger children had increased onset and peak latencies. Adolescents (μ = 15, s = 2) and younger adults (μ = 21, s = 2) typically acquire conditioning quicker than young children and older adults; however, “primary school” children (μ = 7.6, s = 0.50) were more consistent in the expression of CRs compared to younger children (infants and toddlers in 12 month increments), adolescents, and adults (Konrad et al., 2024) Additionally, younger adults (μ = 22.3, s = 2.2) tend to exhibit quicker acquisition than older adults (μ = 64.1, s = 3.8; Cheng et al., 2010). These effects are not exclusive to humans as older rats have been shown to exhibit less CRs than younger rats (Weiss & Thompson, 1991). Participants in Frings et al. (2010) and Coffin et al. (2005) ADHD group had a mean age of 12.3 (s = 1.34) and 9.8 while the control group had a mean age of 12.1 (s = 1.8) and 9.5, respectively. In the single human study found (Gustafsson et al., 2023), the mean age of the ADHD group was 12.5 (s = 2.25). Thus, when employing an EBC procedure to explore cerebellar function in ADHD, it is necessary to primarily compare results between individuals of similar age to avoid confounds.

## 4. Conclusions

Despite the limited number of studies included, there was a wide variety of EBC procedures utilized. While classical conditioning categories and parameters such as trials per day, US duration, and intertrial interval are consistent across experiments involving ADHD models and populations, C/T ratios are not. This lack of standardization could be responsible for mixed findings, as C/T ratio values may engender variations in behavioral outcomes as well as hippocampal recruitment; however, in animal studies, the deficits inherent to each model could also be responsible. Standardization of C/T ratios for studies using EBC paradigms to study cerebellar dysfunction in ADHD, in addition to more human studies, is essential for advancement in this area of research.

## Figures and Tables

**Table 1:** List of included studies, their subjects, and type of EBC procedure used.

Study	Subjects	Conditioning Type
Chess and Green (2008)	SHR vs. Wistar	Delay
Fernández-Lamo et al. (2009)	Hypothyroid vs. Euthyroid	Trace
Green et al. (2011)	SHR vs. Wistar	Delay
Gustafsson et al. (2023)	ADHD children vs. control	Delay
Janke et al. (2015)	SD vs. WKY	Delay
Thanellou and Green (2013)	WKHA vs. WKHT vs. normal Wistar	Delay
Thanellou et al. (2009)	WKHA vs. WKHT	Delay

**Table 2:** Included studies with trials per day, US duration, CS-US interval, average ITI, inter-US interval, and C/T ratio. As a point of comparison, C/T ratios for Coffin and Frings are 63.5 and 69.2 or 26.7 (depending on condition) for Coffin et al. and Frings et al., respectively.

Study	Trials Per Day	US Duration (ms)	CS-US Interval (ms)	ITI (Average)	Inter-US Interval (ms)	C/T Ratio
Chess and Green (2008)	100	15	750	30,000	30,750	41
Fernández-Lamo et al. (2009)	60	0.5	500	30,000	30,500	61
Green et al. (2011)	100	15	475	30,000	30,475	64.2
Gustafsson et al. (2023)	100	100	300 or 500	8000	8300 or 8500	17 or 27.7
Janke et al. (2015)	100	10	490	25,000	25,490	52
Thanellou and Green (2013)	100	15	750	30,000	30,750	41
Thanellou et al. (2009)	100	15	750	30,000	30,750	41

## Data Availability

No new data were created or analyzed in this study.

## Abbreviations

The following abbreviations are used in this manuscript:

EBC	Eyeblink conditioning
ADHD	Attention deficit/hyperactivity disorder
CS	Conditioned stimulus
US	Unconditioned stimulus
CR	Conditioned response
UR	Unconditioned response
SHR	Spontaneously Hypertensive Rat
WKY	Wistar-Kyoto Rat
SD	Sprauge-Dawley
WKHA	Wistar-Kyoto Hyperactive Rat
WKHT	Wistar-Kyoto Hypertensive Rat
VLPFC	ventrolateral prefrontal cortex
DAT-KO	Dopamine Transporter Knock-Out
BDNF	brain-derived neurotrophic factor
ITI	Intertrial interval
ISI	Interstimulus interval
PN	Pontine nuclei
RN	Red nucleus
DAO	Dorsal accessory inferior olive
IPN	Interpositus nucleus
GC	Granule cells
